# Reexamination of honey bee Africanization in Mexico and other regions of the New World

**DOI:** 10.1038/s41598-025-00989-1

**Published:** 2025-05-09

**Authors:** Emeterio Payró de la Cruz, Martina Valencia Domínguez, Rodimiro Ramos Reyes, Adam Tofilski

**Affiliations:** 1 Tecnológico Nacional de México Campus Zona Olmeca, Ocuiltzapotlán, Mexico; 2 El Colegio de La Frontera Sur, Ranchería Guineo, Villahermosa, Mexico; 3 University of Agriculture in Krakow, Krakow, Poland

**Keywords:** *Apis mellifera*, Africanized honey bees, Mexico, Wing, Venation, Adaptive radiation, Biodiversity, Biogeography

## Abstract

Honey bees (*Apis mellifera*) are not native to the New World. The initial introduction of the species to the Americas occurred from Europe, with subsequent introductions from Africa. The African bees hybridized with European bees and are now referred to as Africanized bees. A large feral population was established and subsequently colonized extensive areas of both the North and South American continents, including Mexico. The aim of this study was to conduct a morphometric analysis of geographic variation among Africanized bees. Recently acquired data from Southeastern Mexico were compared with existing datasets of Africanized bees and evolutionary lineages from the Old World. The forewing venation was described using 19 landmarks. The honey bees originating from southeastern Mexico exhibited significant differences from all other investigated populations. It is necessary to verify if the observed geographic variation within Africanized bees is related to natural selection or other factors, including hybridization or genetic drift. Furthermore, honey bees from populations in the USA and Argentina, which appear to have not been affected by Africanization, differed markedly from honey bees naturally occurring in Europe and can be classified as hybrids between evolutionary lineages.

## Introduction

Honey bees (*Apis mellifera*) are native to Europe, Africa, and the Middle East^1^. Within this extensive distribution range, over 26 subspecies have been identified^1^ and later few more subspecies were described^2^. These subspecies have been grouped into four main evolutionary lineages^3,4^. However, recent studies have recognized three additional lineages^5,6^. As a consequence of human intervention, the current distribution of honey bees is significantly more extensive. European settlers introduced them to the American continents on multiple occasions^7,8^. At the early stages of their introduction, the honey bees in question represented European subspecies. However, in 1956, *Apis mellifera scutellata* was introduced from Africa to Brazil^9^. Following their introduction, African bees escaped from experimental hives and hybridized with the previously introduced European bees. These hybrids and their descendants are designated as Africanized bees. They constituted a vast feral population that proliferated from Brazil across extensive regions of both North and South America. At the present time, the range of the Africanized bee extends from the central and southern United States in the north^10,11^ to northern Argentina in the south^12–15^.

The initial detection of Africanized bees in Mexico occurred in 1986 in the state of Chiapas^16,17^. One year later, those bees were observed in nearby Tabasco and Yucatan^18,19^. Their rate of spread was found to be approximately 400 km annually^20^, and by the end of the twentieth century, they had invaded the majority of the country. In northern Mexico, the Africanized bees first appeared in 1989, and by 1993, all colonies had been Africanized^21^. At the present time, the process of Africanization of Mexico’s honey bees appears to have reached a state of relative stability^22^. The proportion of African and European traits varies between regions and is influenced by climate^22^. The prevalence of African traits is greater in warm, semi-dry, and subtropical lowlands. European traits persist in regions with cooler climates and at higher altitudes^20,23^. The humid tropical climate of Tabasco provides an optimal habitat for Africanized bees, and their abundance is significantly higher than in other regions of Mexico^22^. The percentage of Africanized colonies in Tabasco exhibited considerable variation between studies, with estimates ranging from 43.8%^24^ to 100%^22^. Mexico has a high concentration of managed honey bee colonies^25^ and both Africanized and European bees are utilized for beekeeping^22^. Africanized bees demonstrate enhanced tolerance to varroa mites^26^, yet exhibit heightened defensive behaviors in comparison to their European counterparts^27^. Attempts have been made to select the local honey bees for less defensive behavior^28^ and higher honey production^29^.

In the native range of honey bees, isolation by geographic barriers and subsequent genetic drift, as well as natural selection resulted in the formation of the previously mentioned numerous subspecies, which differ in both morphology and behavior^1^. A similar evolutionary trajectory is likely in the case of Africanized bees given their expansive distribution and adaptation to a range of climatic conditions^30,31^. Selective pressures vary across different climatic zones, leading to the emergence of diverse phenotypes in distinct geographic regions^32^. The feral population of Africanized bees is substantial, and after half a century since its introduction, the evolutionary outcomes should be perceptible. In particular, it can be anticipated that populations in humid tropical climates diverged from those in more temperate climates.

There are numerous methods of identification for Africanized honey bees^33^. The initial and most frequently utilized method was based on the measurement of various body parts, including the wings, legs, and sternites^34,35^, which is analogous to the standard morphometry of honey bees^1^. This method is labor-intensive and time-consuming, thus prompting the development of several modifications designed to streamline it^36,37^. The most frequently utilized simplified morphometric identification of Africanized bees is frequently referred to as FABIS^37^. Some simplifications were based on the assumption that Africanized bees are smaller than European honey bees^36,38,39^. One of the methods used to simplify the identification process is geometric morphometrics of the wings^40,41^. This method has been shown to be highly effective, providing a substantial amount of data in a relatively short time frame. The method is based on the coordinates of landmarks and requires only the mounting of wings. The results of wing geometric morphometrics have been found to be in agreement with those of molecular markers^42–44^.

An alternative approach to morphometry is identification based on molecular markers. Mitochondrial DNA has been used to identify Africanized bees^45,46^. However, this method alone is not sufficiently reliable for identifying these bees because it is maternally inherited and does not provide information on the degree of hybridization between African and European ancestors^2^. In addition, European honeybees from the Iberian Peninsula can naturally carry African haplotypes, and significant discrepancies have been observed between identification based on mitochondrial DNA and FABIS^47^. Therefore, it is recommended that molecular identification of Africanized bees should be based on a representative number of nuclear markers. Microsatellites^48^ or single-nucleotide polymorphism^49,50^ may be employed for this purpose. Molecular markers are highly effective, but they are more costly, not accessible to beekeepers, and necessitate more sophisticated methods of sample collection and storage. One advantage of morphometric methods is the potential for comparisons with older data. Specifically, there is a well-established dataset encompassing all four major evolutionary lineages that was collected during periods when human-induced hybridization was minimal^51^.

The objective of this study was to conduct a comparative analysis of honey bees from Tabasco state in Mexico with other populations globally. The results demonstrate that honey bees from southeastern Mexico exhibited significant divergence from other honey bee populations in their native range and on the American continents. This finding is crucial for the identification of Africanized bees and the understanding of their evolution.

## Material and methods

**Fig. 1:**
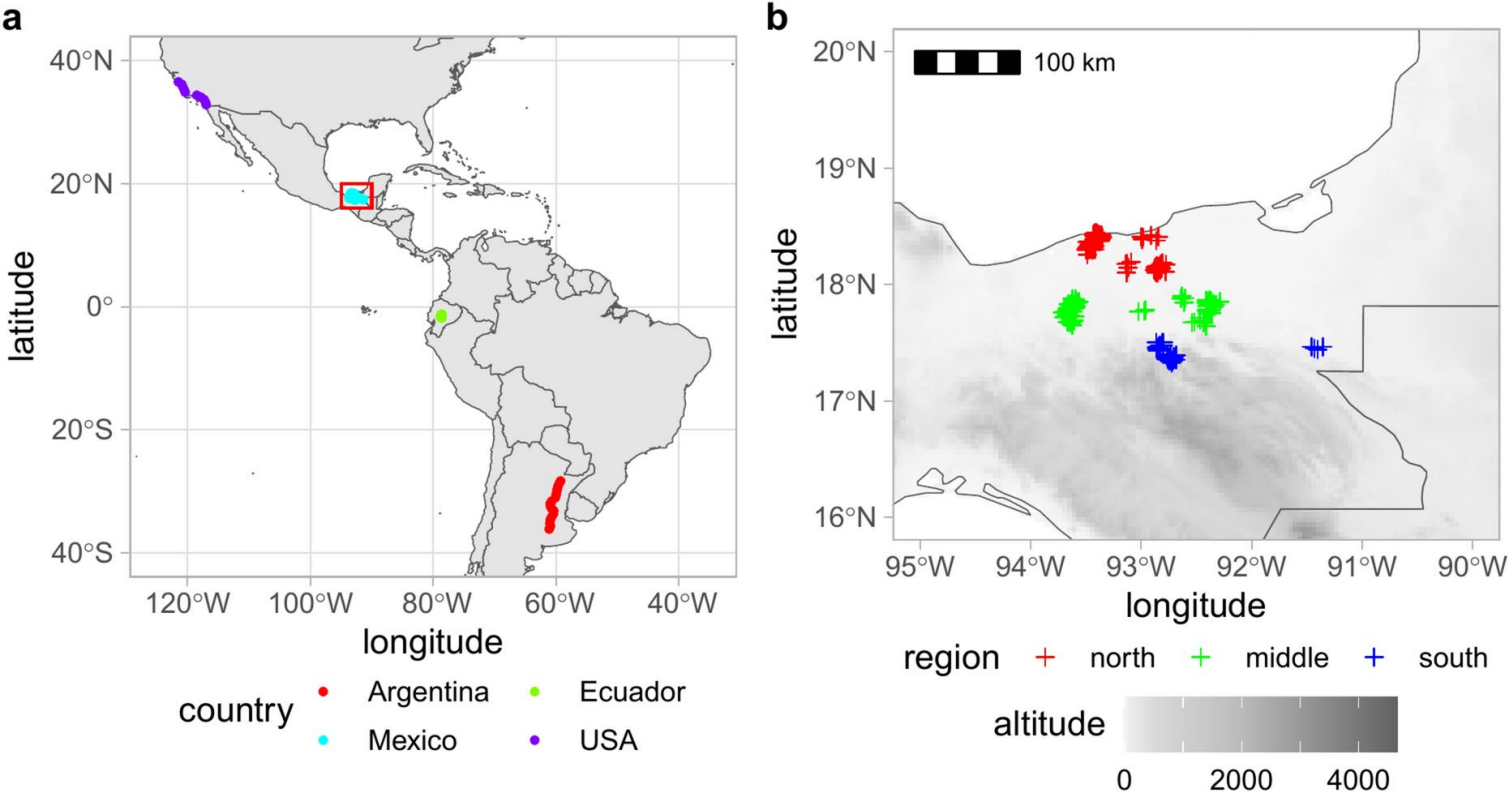
Map of sampling sites in Argentina, Ecuador, Mexico, and the USA (**a**). The Tabasco region in southeastern Mexico, indicated by the red rectangle, has been enlarged (**b**). Maps were generated using data from rnaturalearth v. 0.3.4 (https://CRAN.R-project.org/package=rnaturalearth) and WorldClim (https://worldclim.org/).

A new data set was collected on 2951 honey bee workers originating from 245 colonies located in 33 different sites across three regions of Tabasco state in Mexico (Fig. 1b). The majority of colonies were represented by a sample size of 12 workers. The samples were stored in alcohol. The right forewings were dissected and subsequently dry mounted on microscopic slides. The images of the wings were obtained using a ZEISS Stemi 305 Stereo Microscope, which was equipped with a ZEISS Axiocam 105 microscope camera. Additionally, two publicly available datasets of wing images were utilized: one from Argentina and the USA^38,52^ and the other from Ecuador (Fig. 1a)^53,54^. Argentina and the USA were represented by 177 and 99 wings, respectively. In the case of the two countries, honey bees were collected from flowers along transects, with each location being represented by a single worker. Due to the imprecision associated with morphometric measurements based on a single specimen, the wings from nearby locations were grouped into samples consisting of 10 wings. The number of samples in Argentina and the USA was 18 and 10, respectively. Because the number of wings was not multiple of ten the last sample in Argentina and the USA contained 7 and 9 wings, respectively. Ecuador was represented by 726 wings, 74 colonies and 15 apiaries. As a reference of four evolutionary lineages (A, C, M, and O), 1832 wing images were used to represent 187 colonies and 25 subspecies^51^. These wing images were obtained from the Morphometric Bee Data Bank in Oberursel, Germany and were collected between 1950 and 1995 from the extensive native range of the honey bee, which encompasses Europe, the Middle East, and Africa.

**Fig. 2:**
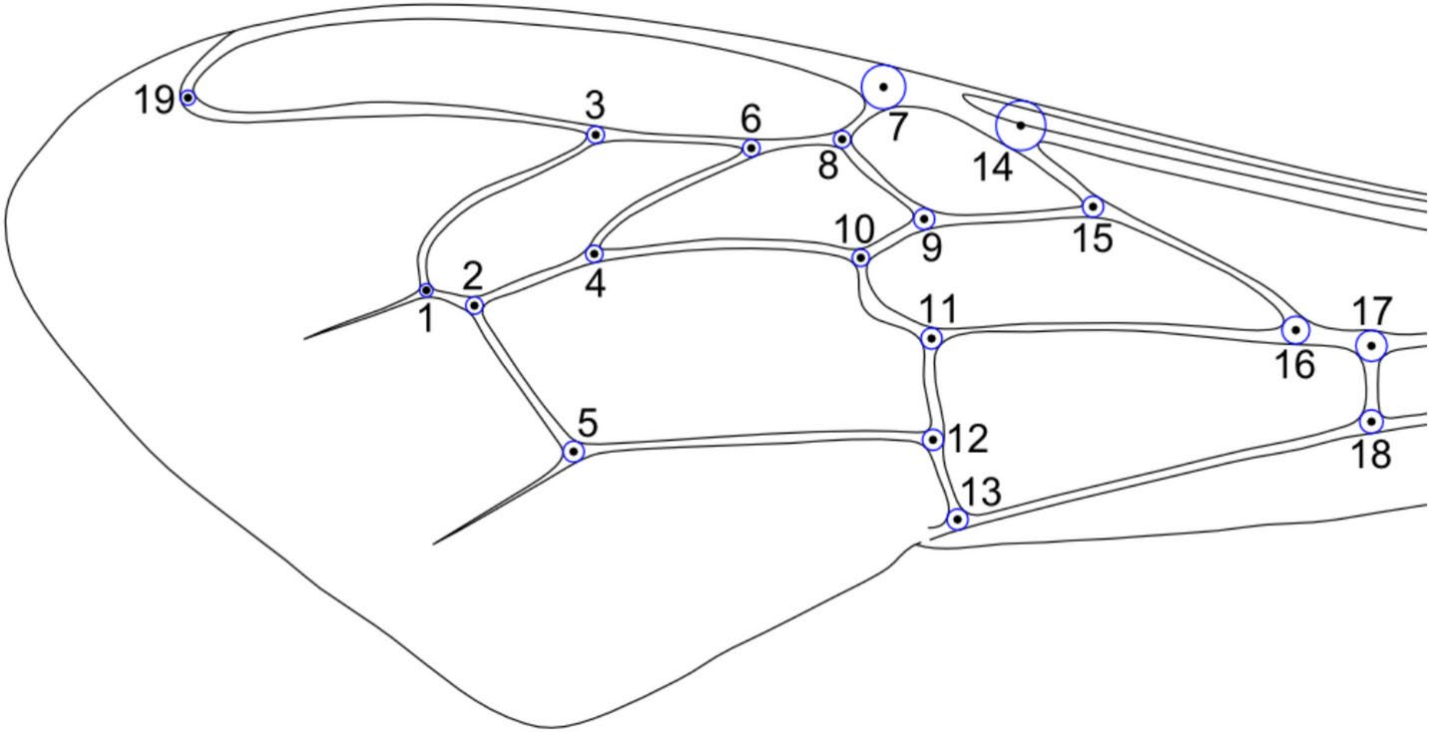
Landmarks marked on a forewing of honey bee worker. The landmarks are indicated with black numbered dots. In order to ensure precise measurements, the blue circles around landmarks should be tangent to the edge of wing venation.

The IdentiFly 1.8 software^51^ was employed to determine 19 landmarks (Fig. 2) on the wing images. The position of the landmarks on the wings from Mexico, Ecuador, Argentina and the USA was determined by a single experienced person. In the case of the linages dataset^51^, the wings were measured by another experienced person.

The statistical analysis was conducted using RStudio version 2023.12.1 and R version 4.3.2. For further details regarding the statistical analysis, please refer to the Supplementary Document S1. The landmark coordinates were aligned using generalized Procrustes analysis implemented in the function “gpagen” from the “geomorph” package^55^. It is important to note that the identification of honey bees to lineages or subspecies based on the measurements of a single wing is not an accurate method^41^. Furthermore, the workers from a single colony are genetically related and not independent of one another. Consequently, the aligned landmark coordinates were averaged within colonies. In the case of honey bees from Argentina and the USA, which were collected from flowers, individual workers were averaged within samples representing nearby locations (Fig. 1a). The colony or sample averages were used in the subsequent statistical analysis. Each landmark was represented by two coordinates, resulting in a total of 38 variables. In the case of multivariate data, ordination is often used to analyze and visualize differences between groups. First, principal component analysis (PCA), which is not based on group membership, was used. As a result of this analysis, a set of principal components was obtained, which were ordered according to their importance. Inspection of the most important principal components can be used to check how different populations overlap. A lack of overlap indicates a clear separation between the groups. Often, differences between populations are present in a large number of principal components. In this case, linear discriminant analysis (LDA) can be used. It uses group membership to maximize the differences between groups. This analysis often separates groups much better than PCA, but there is a risk of artificially inflating the differences between groups when the number of variables is large and the sample size is small. Leave-one-out cross-validation was used to verify the results of LDA. A high percentage of samples correctly identified with cross-validation indicated a clear separation of the groups. To visualize similarities between populations, we used principal coordinates analysis (PCoA), implemented in the function “cmdscale” from the package “stats”. Another method to detect separate populations is cluster analysis. For this purpose, we used model-based clustering implemented in the package “mclust”^56^. The optimal number of clusters was selected using the Bayesian information criterion. To classify the samples as honey bee evolutionary lineages, we used the package “IdentiFlyR”^57^. To test whether wing shape changed with latitude and longitude, we used multivariate regression implemented in the function “procD.lm” from the package “geomorph”^55^. Differences in wing shape between populations were tested using multivariate analysis of variance, implemented in the function “manova” from the package “stats”. Most of the statistical analysis was based on the first 34 principal components. The last four components were not used because their eigenvalues are zero after Procrustes analysis^58^. Only in the case of cluster analysis, the first 18 principal components explaining 95% of the variance were used. The number of principal components used was reduced to increase the stability of the results.

## Results

**Fig. 3:**
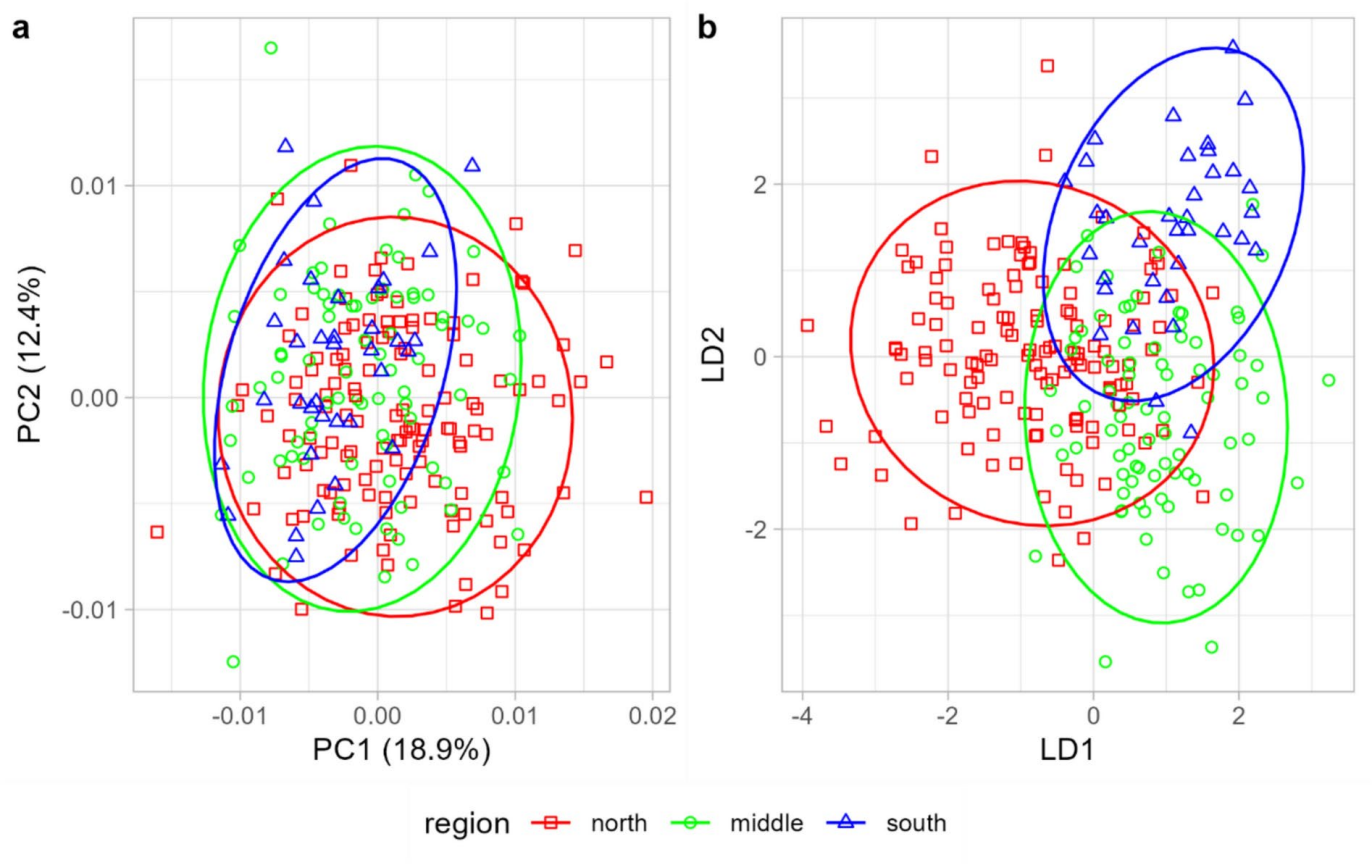
The variation in wing shape across three regions of Mexico is illustrated by the first two principal components (**a**) or the first two linear discriminants (**b**). Ellipses represent 95% confidence regions.

The shape of honey bee wings from Mexican samples varied significantly with latitude and to a lesser extent with longitude (multivariate regression, latitude: F = 9.94, P = 0.001; longitude: F = 2.13, P = 0.015). When these bees were arbitrarily divided into three groups: northern, middle, and southern regions, there was considerable overlap between them in the plot of the first two principal components (Fig. 3a). The plot of the first two linear discriminant functions showed a more pronounced separation of the three groups, but some overlap remained (Fig. 3b). The three groups could not be completely discriminated. The classification success rates (with leave-one-out cross-validation) for the northern, middle, and southern regions were 73.4, 73.1, and 85.7%, respectively.

**Fig. 4:**
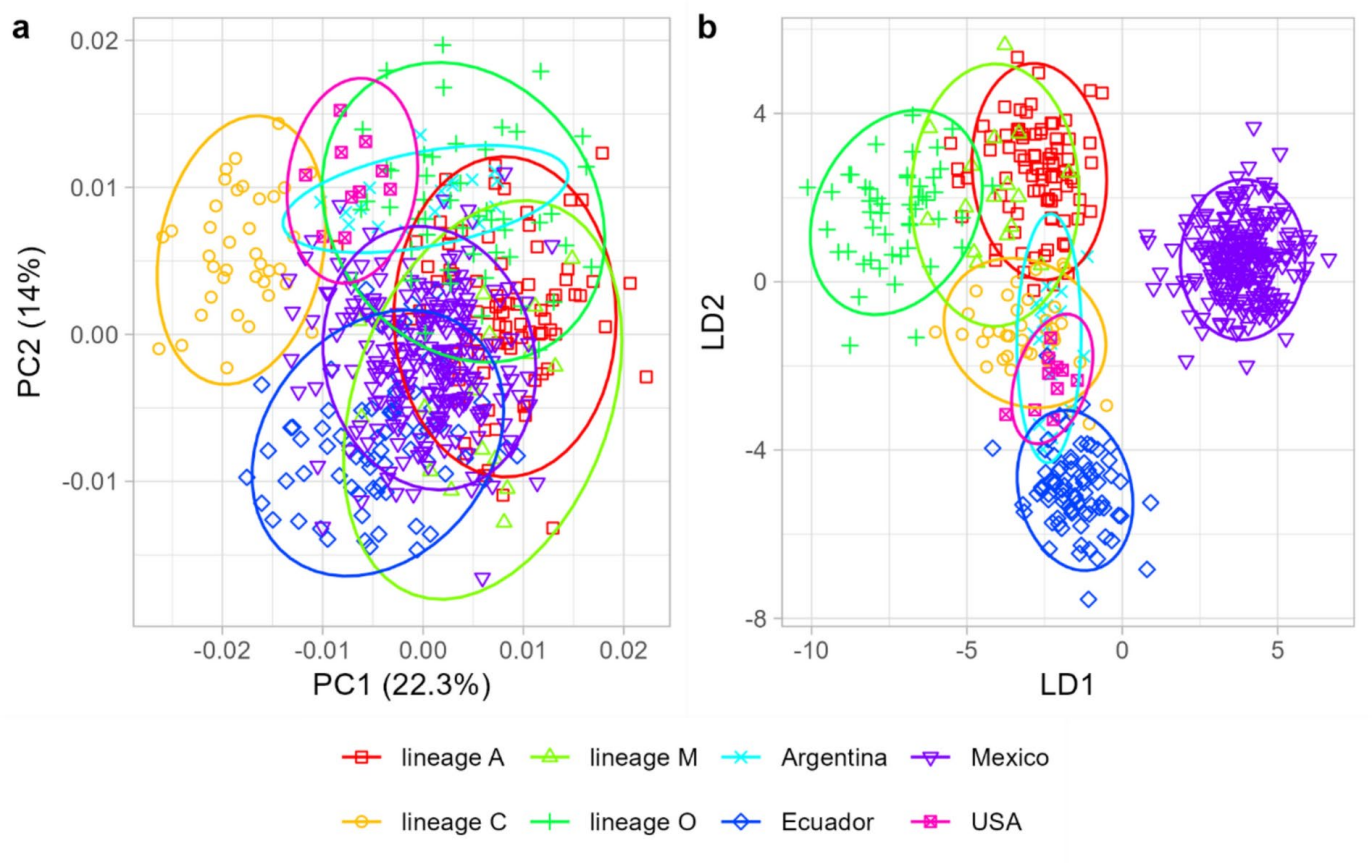
The variation in wing shape among honey bee populations from various parts of the world illustrated by the first two principal components (**a**) or the first two linear discriminants (**b**). Ellipses represent 95% confidence regions.

**Fig. 5:**
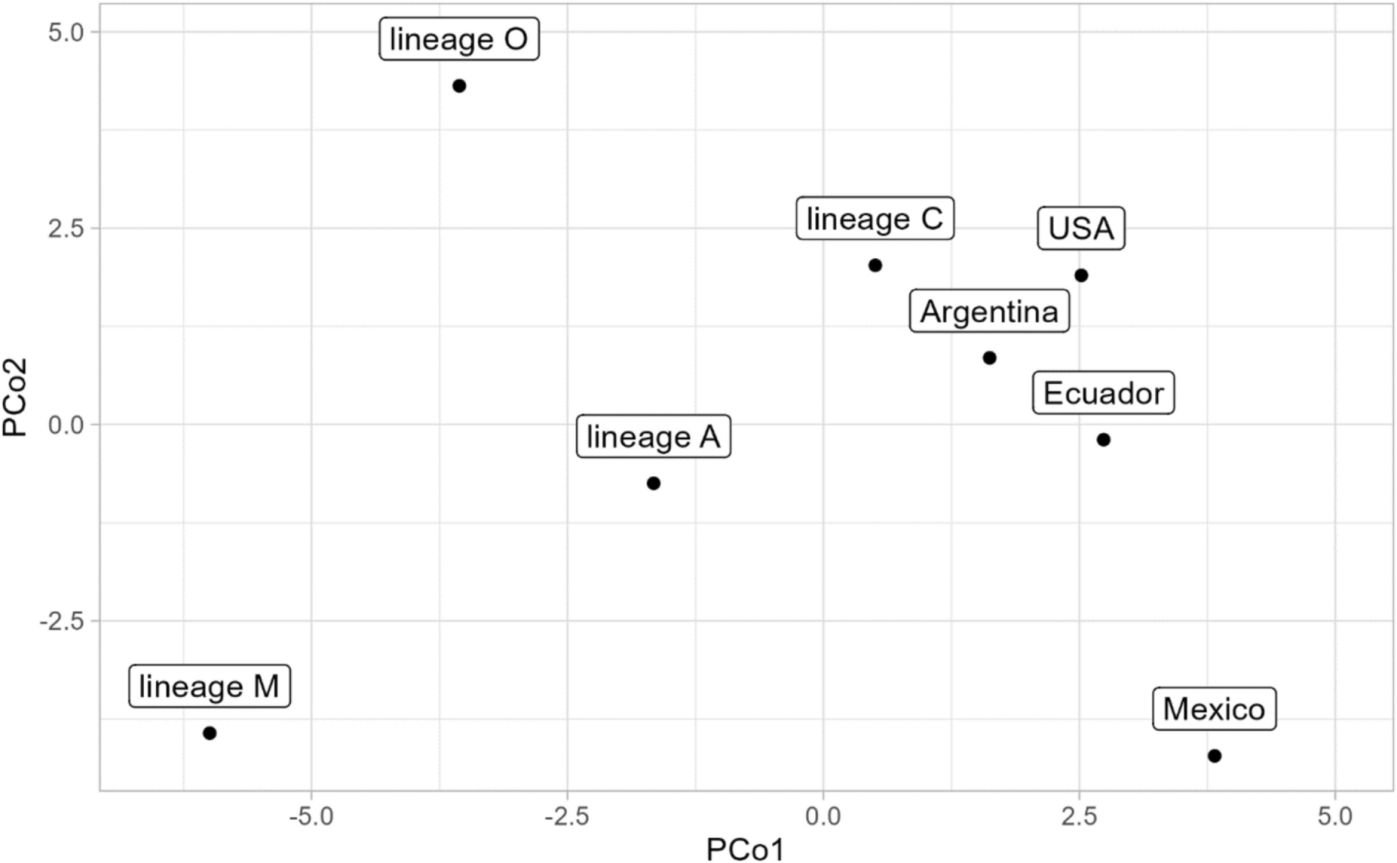
The first two principal coordinates calculated from pairwise Mahalanobis distances between populations.

A significant divergence in wing shape was observed among populations (Fig. 4a,b) comprising countries from the New World (Ecuador, Argentina, Mexico, and the USA) and the Old World evolutionary lineages (A, C, M, and O) (multivariate analysis of variance: F = 22.18, P < 10^–15^). Pairwise comparisons revealed no significant differences between Argentina and the USA (Table 1); all other populations differed significantly from each other (Table 1). New World populations diverged markedly from Old World populations, and the divergence was comparable to or larger than the differences between Old World lineages (Fig. 5). Honey bees from Mexico differed markedly from all other populations and could be identified (with leave-one-out cross-validation) without error. Honey bees from Ecuador also differed markedly, but in this case, a single colony (1.35%) was misclassified as Argentina. The majority of misclassifications occurred between honey bees from Argentina and the USA (Table 2), with five out of 13 samples from Argentina being incorrectly classified as belonging to the USA.

**Table 1:** The differences between honey bee populations in wing shape (expressed as Mahalanobis distances, lower triangle) and significance of pairwise comparisons (upper triangle).

Population	Lineage A	Lineage C	Lineage M	Lineage O	Argentina	Ecuador	Mexico	USA
Lineage A	–	0.0001	0.0001	0.0001	0.0001	0.0001	0.0001	0.0001
Lineage C	8.31	–	0.0001	0.0001	0.0001	0.0001	0.0001	0.0001
Lineage M	7.72	10.52	–	0.0001	0.0001	0.0001	0.0001	0.0001
Lineage O	6.36	9.02	9.93	–	0.0001	0.0001	0.0001	0.0001
Argentina	6.17	8.19	9.56	7.98	–	0.0001	0.0001	0.8345
Ecuador	7.78	8.74	10.43	9.42	6.16	–	0.0001	0.0001
Mexico	7.30	9.47	11.09	11.50	7.75	7.91	–	0.0001
USA	6.94	7.31	10.56	8.11	2.45	5.95	8.01	–

**Table 2:** Confusion matrix for classification of honey bee populations.

population	Lineage A	Lineage C	Lineage M	Lineage O	Argentina	Ecuador	Mexico	USA
Lineage A	84	0	0	0	1	0	0	0
Lineage C	0	37	0	0	0	0	0	0
Lineage M	0	0	16	0	0	0	0	0
Lineage O	1	0	0	48	0	0	0	0
Argentina	0	0	0	0	13	0	0	5
Ecuador	0	0	0	0	1	73	0	0
Mexico	0	0	0	0	0	0	245	0
USA	0	0	0	0	1	0	0	9

**Fig. 6:**
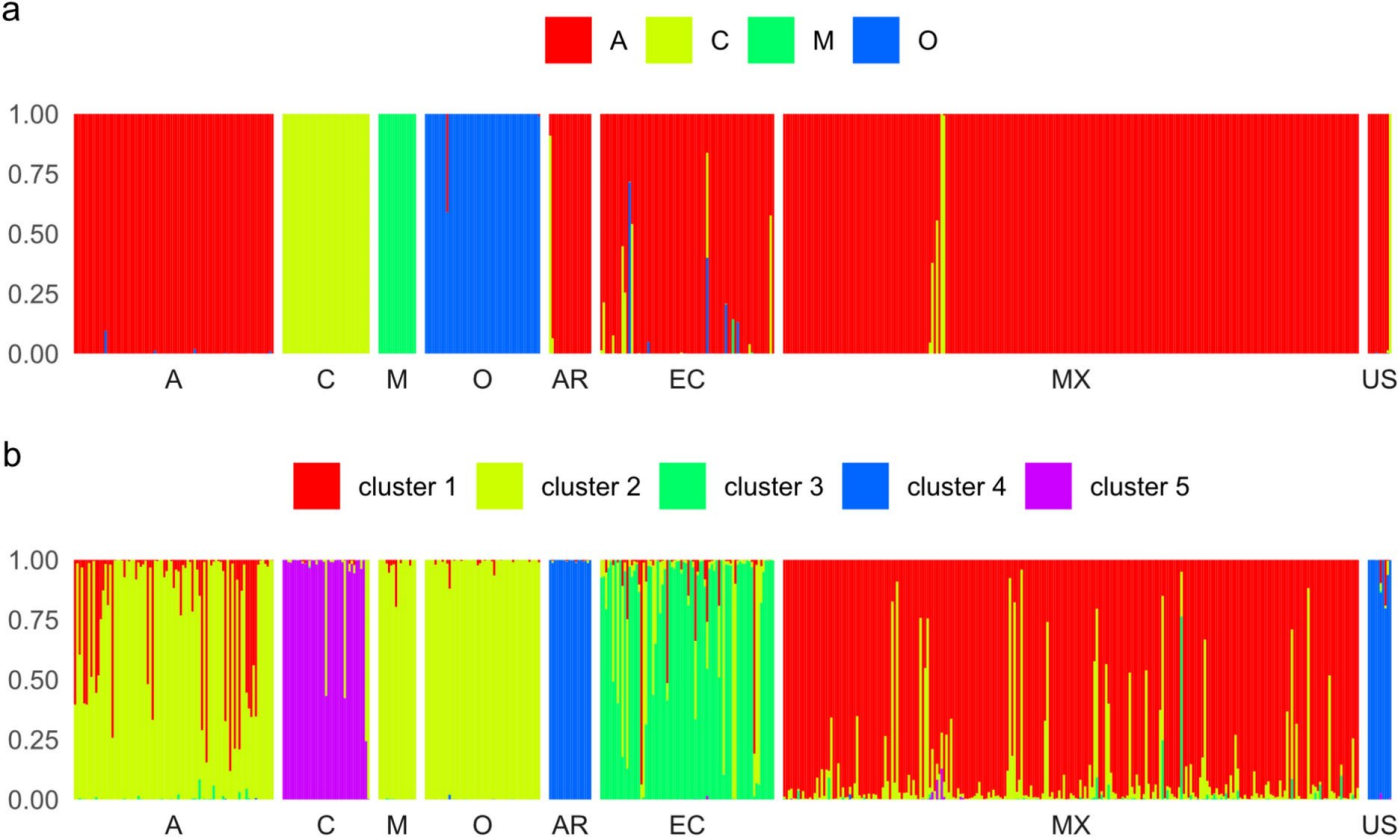
The classification of reference datasets (lineages: A, C, M, O) and samples from Argentina (AR), Ecuador (EC), Mexico (MX), and the USA (US) as four evolutionary lineages (**a**) or five clusters detected by model-based clustering (**b**). The columns represent classification probabilities, which were rescaled so that their sum for each sample equals one.

When the mean values of wing shape in samples from Argentina, Ecuador, Mexico, and the USA were used as unknowns and classified as one of four evolutionary lineages, 97.4% of them were classified as lineage A (Fig. 6a). In Argentina, the single sample classified as lineage C originated from the most southern location of the transect. In Ecuador, three samples were classified as lineage C, and one sample was classified as lineage O. No clear geographical pattern in the location of samples classified as lineages other than lineage A was observed in Ecuador. In Mexico, all three colonies classified as lineage C originated from a single location in the northern part of Tabasco. In the USA, the only sample classified as lineage C originated from the most northern location of the transect (for details, see Supplementary Document 1).

The application of cluster analysis (without prior information about ancestral populations) led to the distinction of five clusters that agreed more closely with recent sample location than with the assignment to old world lineages. Cluster 1 primarily comprised Mexican honey bees, with some samples from Ecuador and lineage A. Cluster 2 consisted of samples from all Old World lineages, with some samples from Ecuador and Mexico. Cluster 3 predominantly contained samples from Ecuador, with some from Mexico. Cluster 4 contained all samples from both Argentina and the USA. Finally, Cluster 5 exclusively comprised samples from lineage C (Fig. 6b).

## Discussion

The data presented here unequivocally demonstrate substantial geographic variation of Africanized bees, both in local contexts (Fig. 3) and at broader geographic scales (Fig. 4). Although Mexican honey bees exhibited the greatest similarity to lineage A, they demonstrated clear differences from it (Fig. 4b, Table 1). The divergence between the Mexican population and the four lineages was remarkably pronounced, reaching a level comparable to that observed between the lineages themselves (Table 2). Indeed, lineage A exhibited a greater divergence from the Mexican honey bees than it did from lineages M and O (Table 2). As indicated by earlier studies, hybrids manifest an intermediate phenotype between the parental populations^59,60^. However, the phenotype of the Mexican honey bees was distinctly separated from the other populations and positioned well beyond the midpoint between lineage A and C or M, particularly in the graph of the first two linear discriminants (Fig. 4b). Earlier studies that employed genetic markers reported analogous differences between Africanized bee populations^61–63^. Furthermore, some Africanized populations exhibit substantial variation in behavioral traits^64^.

The observed discrepancies between Mexican honey bees and lineage A can be attributed to the founder effect. The initial population introduced from Africa to Brazil in 1956 was relatively small, and the introduced honey bees carried only a portion of the original variation present in lineage A. Furthermore, the honey bees introduced from Africa hybridized with the European bees that had been introduced earlier, resulting in a combination of phenotypes from both origins^59^. However, at the early stage of Africanization, the dispersing population was presumably relatively homogenous in terms of genetics and morphology. The observed variations among Africanized bees from Argentina, Ecuador, Mexico, and the USA (Figs. 4, 5) emerged subsequently and can be attributed to natural selection, hybridization with European managed populations or genetic drift. It is particularly intriguing to consider the possibility that a substantial portion of the observed variation among Africanized bee populations may be attributable to natural selection. In the native range of honey bees, natural selection has been documented to result in the formation of numerous subspecies^1^. A similar evolutionary trajectory is plausible for Africanized bees, given their extensive distribution across diverse climatic conditions^31^. The differentiation of Mexican honey bees may be further enhanced by geographic isolation. The Isthmus of Panama represents a significant barrier to gene flow from South America to Central America. Additionally, the mountain ranges in the central region of Mexico act as a barrier to gene flow from the northern to the southern part of the country. The presence of natural selection and adaptation in Africanized bees has been previously documented^38,63,65^. Rinderer and Hellmich^32^ predicted that “the possibilities exist for natural selection to produce, rather rapidly, new ecotypes of honey bees in the Americas.” The distinctness of honey bee populations from Mexico and Ecuador is consistent with this prediction; however, the present study cannot exclude the possibility that differentiation was related to hybridization or genetic drift^66^.

In addition to the differentiation of American honey bee populations, we identified potential trends towards uniformity related to beekeeping practices. Our findings revealed a surprising degree of similarity between honey bees from the USA and Argentina^38^. This similarity may be attributed to the exchange of genetic material between beekeepers. It is noteworthy that the samples from the USA and Argentina exhibit notable divergence from lineage C and M (Table 1), which are naturally prevalent in Europe. Furthermore, they exhibit greater similarity to lineage A (Table 1). The presence of some similarity between lineage A does not necessarily indicate that the honey bees are of Africanized origin, as hybrids can be classified as lineage A even if they differ markedly from it^67^. The data presented here suggest that samples from the northern regions of the USA and southern regions of Argentina should be classified as hybrids rather than as originating from Europe. The hybridization of commercial stock of honey bees in the Americas can be either local or have occurred during the last 30 years, as evidenced by the fact that European samples obtained from Venezuela by Rinderer et al.^59^ were similar to lineage C. However, it should be noted that more data from the USA and Argentina is needed to reach a more definitive conclusion about the hybridization of those populations.

It had previously been posited that “wing venation patterns can discriminate distantly related species of *Apis* but are not useful at the population level within species”^62^. However, the data presented here demonstrate that wing measurements can be utilized to detect differences between populations of Africanized bees and to compare them with evolutionary lineages from the Old World. Indeed, the evolutionary lineages were initially delineated through morphometric methodologies^1^. There is a wealth of historical data concerning the biogeography of honey bees. These historical morphometric data can be utilized to validate genetic markers in cases where the provenance of a sample is ambiguous, whether it is native to a particular region or introduced by beekeepers. The morphometric identification of Africanized bees can be improved by increasing the size and extent of reference samples, thereby enhancing the precision and reliability of the identification process. The availability of reference data from other American populations would facilitate not only the more reliable discrimination between Africanized and European honey bees but also the indication of their region of origin. The identification of Africanized bees based on geometric morphometrics of the wing is a more time-efficient and less labor-intensive process than FABIS. The reference material of Africanized bees used in FABIS originated from Argentina, Brazil, Guyana, Surinam, and French Guiana^68^. The data presented here demonstrate not only the presence of geographic variation within Africanized bees but also that bees used by beekeepers can differ from those used in the 1970s, when FABIS was created. Consequently, there is a necessity to update this method.

## Conclusions

The present study demonstrates that honey bees from southeastern Mexico and Ecuador are distinctly different from other populations around the world. Additionally, it can be hypothesized that honey bees utilized by beekeepers in the American continents may have also undergone changes in recent years, becoming less similar to European honey bees. These findings are crucial for the accurate identification of Africanized bees.

## Supplementary Information

Supplementary Information.

## Data Availability

Images of the fore wings of honey bees collected in Tabasco, Mexico, along with other relevant data, including landmark coordinates and geographic coordinates of sampling locations, are available for download from 10.5281/zenodo.13884732. Landmark coordinates associated with the wing images provided by Calfee et al.^52^ are available for download from https://doi.org/10.5281/zenodo.13884183. Landmark coordinates associated with the wing images provided by Masaquiza and Arenal^54^ are available for download from https://doi.org/10.5281/zenodo.14604363. A statistical analysis of the data is presented as an R script in Supplementary Document 1.
