# Supplementary material for: Reexamination of honey bee Africanization in Mexico and other regions of the New World
Source: Sci Rep. 2025 May 9;15:16267. doi: 10.1038/s41598-025-00989-1 (PMC12064644; doi:10.1038/s41598-025-00989-1)
Supplement: Supplementary file 1 — Supplementary Information. [file 41598_2025_989_MOESM1_ESM.html]

Reexamination of honey bee Africanization in Mexico and other regions of the New World


# Reexamination of honey bee Africanization in Mexico and other regions of the New World

Supplementary document with statistical analysis

Authors

Affiliations

Emeterio Payró de la Cruz

Tecnológico Nacional de México campus Zona Olmeca, Mexico

Martina Valencia Domínguez

Tecnológico Nacional de México campus Zona Olmeca, Mexico

Rodimiro Ramos Reyes

El Colegio de la Frontera Sur, Mexico

Adam Tofilski

University of Agriculture in Krakow, Poland

Published

2025-01-07

# Libraries

```
# calculations
library(geomorph)  # GPA 
library(Morpho)  # CVA
library(IdentiFlyR)  # classification
library(dplyr)  # left_join
library(mclust)  # clustering

# plotting and visualization
library(ggplot2)  # plots
ggplot2::theme_set(theme_light())
library(rnaturalearth)  # maps
library(raster)  # raster
library(ggspatial)  # annotation_scale
library(ggpubr)  # ggarange
library(reshape2)  # melt
```

# Variable names

```
p <- 19  # number of landmarks
k <- 2  # number of dimensions, in this case 2 for coordinates (x, y)

# create coordinates names used by IdentiFly
xyNames <- c("x1", "y1")
for (i in 2:p) {
  xyNames <- c(xyNames, paste0("x", i))
  xyNames <- c(xyNames, paste0("y", i))
}
xyNames
```

```
 [1] "x1"  "y1"  "x2"  "y2"  "x3"  "y3"  "x4"  "y4"  "x5"  "y5"  "x6"  "y6" 
[13] "x7"  "y7"  "x8"  "y8"  "x9"  "y9"  "x10" "y10" "x11" "y11" "x12" "y12"
[25] "x13" "y13" "x14" "y14" "x15" "y15" "x16" "y16" "x17" "y17" "x18" "y18"
[37] "x19" "y19"
```

```
# The number of principal components used is 2*p-4 = 34, which is equal to the
# degrees of freedom
pcNames <- paste0("PC", 1:(2 * p - 4))
pcNames
```

```
 [1] "PC1"  "PC2"  "PC3"  "PC4"  "PC5"  "PC6"  "PC7"  "PC8"  "PC9"  "PC10"
[11] "PC11" "PC12" "PC13" "PC14" "PC15" "PC16" "PC17" "PC18" "PC19" "PC20"
[21] "PC21" "PC22" "PC23" "PC24" "PC25" "PC26" "PC27" "PC28" "PC29" "PC30"
[31] "PC31" "PC32" "PC33" "PC34"
```

```
# define which landmarks are connected by lines in wireframe graph
link.x <- c(1, 1, 2, 2, 3, 3, 4, 4, 5, 6, 7, 7, 7, 8, 9, 9, 10, 11, 11, 12, 13, 14,
  15, 16, 17)
link.y <- c(2, 3, 4, 5, 6, 19, 6, 10, 12, 8, 8, 14, 19, 9, 10, 15, 11, 12, 16, 13,
  18, 15, 16, 17, 18)
links.apis <- cbind(link.x, link.y)

GP1 <- gridPar(pt.bg = "blue", link.col = "blue", pt.size = 1, tar.pt.bg = "red",
  tar.link.col = "red")
```

# Read data

## Read original data from Mexico

The wing images and their measurements are available at Zenodo (Payró de la Cruz et al. 2024).

```
xyRaw <- read.csv("https://zenodo.org/record/13884732/files/MX-raw-coordinates.csv")
geoData <- read.csv("https://zenodo.org/record/13884732/files/MX-data.csv")
```

## Read data from Argentina and USA

The data are related to study by Calfee et al. (2020a). Wing images are available at Dryad (Calfee et al. 2020b) and wing measurements are available at Zenodo (Tofilski 2025a).

```
xyRaw <- rbind(xyRaw, read.csv("https://zenodo.org/record/14604479/files/AR-raw-coordinates.csv"))
xyRaw <- rbind(xyRaw, read.csv("https://zenodo.org/record/14604479/files/US-raw-coordinates.csv"))
geoData <- rbind(geoData, read.csv("https://zenodo.org/record/14604479/files/AR-data.csv"))
geoData <- rbind(geoData, read.csv("https://zenodo.org/record/14604479/files/US-data.csv"))
```

## Read data from Ecuador

The data are related to study by Masaquiza et al. (2024). Wing images are available at Zenodo (Masaquiza & Arenal 2024) and wing measurements are available at Zenodo (Tofilski 2025b).

```
xyRaw <- rbind(xyRaw, read.csv("https://zenodo.org/record/14604363/files/EC-raw-coordinates.csv"))
geoData <- rbind(geoData, read.csv("https://zenodo.org/record/14604363/files/EC-data.csv"))

# combine landmark coordinates with geographic data
geoData[, c("file", "date", "resolution", "notes")] <- NULL  # remove columns
geoData$group <- substr(geoData$sample, 1, 2)
xyRaw <- cbind(xyRaw, geoData)

geoDataSample <- aggregate(geoData[c("latitude", "longitude")], by = list(geoData$sample),
  FUN = mean)
geoDataSample <- data.frame(geoDataSample, row.names = 1)  # move column 1 to row names
geoDataSample$country <- substr(rownames(geoDataSample), 1, 2)
geoDataSample$country <- gsub("AR", "Argentina", geoDataSample$country)
geoDataSample$country <- gsub("EC", "Ecuador", geoDataSample$country)
geoDataSample$country <- gsub("MX", "Mexico", geoDataSample$country)
geoDataSample$country <- gsub("US", "USA", geoDataSample$country)
```

## Read data from evolutionary lineages

The data are related to study by Nawrocka et al., (2018a). Wing measurements are available at Zenodo Nawrocka et al., (2018b).

```
xyACMO <- read.csv("https://zenodo.org/record/7567336/files/Nawrocka_et_al2018.csv")
xyACMO$sample <- substr(xyACMO$file, 1, 10)

geoDataACMO <- read.csv("https://zenodo.org/record/7567336/files/Nawrocka_et_al2018-geo-data.csv")
tmp <- data.frame(sample = geoDataACMO$sample, latitude = geoDataACMO$latitude, longitude = geoDataACMO$longitude,
  group = geoDataACMO$lineage)

xyACMO <- left_join(xyACMO, tmp, by = "sample")
xyRaw <- rbind(xyRaw, xyACMO)

# number of wings per population
table(xyRaw$group)
```

```
   A   AR    C   EC    M   MX    O   US 
 823  177  369  726  158 2951  482   99
```

## Maps

```
# Read elevation data In order to download the TIF file uncomment the two lines
# below
# download.file('https://geodata.ucdavis.edu/climate/worldclim/2_1/base/wc2.1_2.5m_elev.zip',
# 'wc2.1_2.5m_elev.zip') unzip('wc2.1_2.5m_elev.zip') or use your local file
altRaster <- raster("D:/WorldClim/wc2.1_2.5m_elev.tif")
altPalette <- colorRampPalette(c("#f7f7f7", "#f0f0f0", "#d9d9d9", "#bdbdbd", "#636363"),
  bias = 3)
x.min <- -95
x.max <- -90
y.min <- 16
y.max <- 20
e <- extent(x.min - 5, x.max + 5, y.min - 5, y.max + 5)
altRasterCrop <- crop(altRaster, e)
altDF <- data.frame(rasterToPoints(altRasterCrop))
colnames(altDF) <- c("longitude", "latitude", "altitude")

world <- ne_countries(scale = "medium", returnclass = "sf")

mapA <- ggplot(data = world) + geom_sf() + coord_sf(xlim = c(-125, -35), ylim = c(-40,
  40)) + annotate("rect", xmin = x.min, xmax = x.max, ymin = y.min, ymax = y.max,
  alpha = 0, color = "red") + geom_point(data = geoDataSample, aes(x = longitude,
  y = latitude, color = country), size = 0.8) + scale_color_manual(name = "country",
  values = rainbow(4)) + theme(legend.position = "bottom")
mapA
```

```
MXgeoData <- geoDataSample[geoDataSample$country == "Mexico", ]
MXgeoData$region <- ifelse(MXgeoData$latitude > 18, "north", ifelse(MXgeoData$latitude <
  17.5, "south", "middle"))
MXgeoData$region <- factor(MXgeoData$region, levels = c("north", "middle", "south"))  #specify order
# sample size per region
table(MXgeoData$region)
```

```
 north middle  south 
   128     82     35
```

```
mapB <- ggplot(data = world) + geom_raster(data = altDF, aes(longitude, latitude,
  fill = altitude)) + scale_fill_gradientn(colours = altPalette(100)) + geom_sf(fill = NA) +
  geom_jitter(data = MXgeoData, shape = 3, aes(x = longitude, y = latitude, colour = region),
    width = 0.04, height = 0.04) + coord_sf(xlim = c(x.min, x.max), ylim = c(y.min,
  y.max)) + scale_color_manual(name = "region", values = rainbow(3)) + annotation_scale(location = "tl",
  width_hint = 0.2) + theme(legend.position = "bottom")
mapB
```

```
ggarrange(mapA, mapB, labels = c("a", "b"), font.label = list(size = 12, face = "bold"),
  ncol = 2, nrow = 1)
```

```
# ggsave('map.pdf', width = 170, height = 107, units = 'mm') ggsave('map.png',
# width = 170, height = 107, units = 'mm')
```

## GPA-alignment

```
# Convert from 2D array to 3D array
xy3D <- arrayspecs(xyRaw[xyNames], p, k)
dimnames(xy3D)[[3]] <- xyRaw$file

# Align the coordinates using Generalized Procrustes Analysis
GPA <- gpagen(xy3D, print.progress = FALSE)
consensus <- GPA$consensus

# plot landmarks after alignment
plotAllSpecimens(GPA$coords, links = links.apis, label = TRUE, plot.param = list(pt.bg = "black",
  pt.cex = 0.5, mean.bg = "red", mean.cex = 1, link.col = "red", txt.pos = 3, txt.cex = 1))
```

```
# Convert from 3D array to 2D array
xyAligned <- as.data.frame(two.d.array(GPA$coords))
colnames(xyAligned) <- xyNames
xyAligned$latitude <- xyRaw$latitude
xyAligned$longitude <- xyRaw$longitude

xySample <- aggregate(xyAligned, by = list(xyRaw$sample), FUN = mean)
xySample <- data.frame(xySample, row.names = 1)  # move column 1 to row names
xySample$group <- substr(rownames(xySample), 1, 2)
xySample$group <- gsub("AR", "Argentina", xySample$group)
xySample$group <- gsub("EC", "Ecuador", xySample$group)
xySample$group <- gsub("MX", "Mexico", xySample$group)
xySample$group <- gsub("US", "USA", xySample$group)
xySample$group <- gsub("A-", "lineage A", xySample$group)
xySample$group <- gsub("C-", "lineage C", xySample$group)
xySample$group <- gsub("M-", "lineage M", xySample$group)
xySample$group <- gsub("O-", "lineage O", xySample$group)
xySample$group <- factor(xySample$group, levels = c("lineage A", "lineage C", "lineage M",
  "lineage O", "Argentina", "Ecuador", "Mexico", "USA"))  #specify order

# number of samples per population
table(xySample$group)
```

```
lineage A lineage C lineage M lineage O Argentina   Ecuador    Mexico       USA 
       85        37        16        49        18        74       245        10
```

# Comparison between regions of Mexico

## PCA of Mexican samples

```
MXsample <- xySample[xySample$group == "Mexico", c(xyNames, "latitude", "longitude")]

PCAmx <- prcomp(MXsample[, xyNames])
PCAmx.scores <- as.data.frame(PCAmx$x)
PCAmx.scores$region <- MXgeoData$region

# create plot labels
variance.tab <- summary(PCAmx)$importance
variance <- variance.tab["Proportion of Variance", "PC1"]
variance <- round(100 * variance, 1)
label.x <- paste0("PC1 (", variance, "%)")
variance <- variance.tab["Proportion of Variance", "PC2"]
variance <- round(100 * variance, 1)
label.y <- paste0("PC2 (", variance, "%)")

figMXa <- ggplot(PCAmx.scores, aes(x = PC1, y = PC2, shape = region, color = region)) +
  geom_point() + scale_shape_manual(name = "region", values = c(0:2)) + scale_color_manual(name = "region",
  values = rainbow(3)) + stat_ellipse() + xlab(label.x) + ylab(label.y)
figMXa
```

## Multivariate regresssion of wing shape in Mexico

```
# Convert 2D array into a 3D array
MX3D <- arrayspecs(MXsample[xyNames], p, k)
MXgdf <- geomorph.data.frame(coords = MX3D, latitude = MXsample$latitude, longitude = MXsample$longitude)
fit <- procD.lm(coords ~ latitude + longitude, data = MXgdf, print.progress = FALSE)
anova(fit)$table
```

```
           Df     SS       MS   Rsq    F    Z Pr(>F)   
latitude    1 0.0017 0.001717 0.039 9.94 6.11  0.001 **
longitude   1 0.0004 0.000369 0.008 2.13 2.13  0.015 * 
Residuals 242 0.0418 0.000173 0.952                    
Total     244 0.0439                                   
---
Signif. codes:  0 '***' 0.001 '**' 0.01 '*' 0.05 '.' 0.1 ' ' 1
```

## Linear discriminant analysis of regions in Mexico

```
# use equal prior probability for all groups
n.gr <- length(unique(PCAmx.scores$region))  # number of groups
sample.cva <- CVA(PCAmx.scores[pcNames], PCAmx.scores$region, rounds = 10000, cv = TRUE,
  prior = rep(1/n.gr, n.gr))
sample.cva.scores <- as.data.frame(sample.cva$CVscores)
# rename variable names from CV to LD otherwise use `CV 1`
colnames(sample.cva.scores) <- gsub("CV ", "LD", colnames(sample.cva.scores))
sample.cva.scores$region <- PCAmx.scores$region

figMXb <- ggplot(sample.cva.scores, aes(x = LD1, y = LD2, shape = region, color = region)) +
  geom_point() + scale_shape_manual(name = "region", values = c(0:2)) + scale_color_manual(name = "region",
  values = rainbow(3)) + stat_ellipse()
figMXb
```

```
ggarrange(figMXa, figMXb, labels = c("a", "b"), font.label = list(size = 12, face = "bold"),
  ncol = 2, nrow = 1, common.legend = TRUE, legend = "bottom")
```

```
# ggsave('PCA-LDA-MX.pdf', width = 170, height = 107, units = 'mm')
# ggsave('PCA-LDA-MX.png', width = 170, height = 107, units = 'mm')

# Confusion matrix
CVA.class <- typprobClass(sample.cva$CVscores, groups = as.factor(sample.cva.scores$region),
  outlier = 0)
print(CVA.class)
```

```
 cross-validated classification results in frequencies
        
         middle north south
  north      24    94    10
  middle     60    10    12
  south       4     1    30


 cross-validated classification result in %
        
          middle   north   south
  north  18.7500 73.4375  7.8125
  middle 73.1707 12.1951 14.6341
  south  11.4286  2.8571 85.7143


 overall classification accuracy: 75.10204 %

 Kappa statistic: 0.61011
```

```
# Mahalanobis distances between groups
sample.cva$Dist$GroupdistMaha
```

```
       north middle
middle  1.93       
south   2.44   2.22
```

# Comparison between populations

## PCA of all samples

```
PCA <- prcomp(xySample[, xyNames])
PCA.scores <- as.data.frame(PCA$x)
PCA.scores$group <- xySample$group

# create plot labels
variance.tab <- summary(PCA)$importance
variance <- variance.tab["Proportion of Variance", "PC1"]
variance <- round(100 * variance, 1)
label.x <- paste0("PC1 (", variance, "%)")
variance <- variance.tab["Proportion of Variance", "PC2"]
variance <- round(100 * variance, 1)
label.y <- paste0("PC2 (", variance, "%)")

figPCAa <- ggplot(PCA.scores, aes(x = PC1, y = PC2, shape = group, color = group)) +
  geom_point() + scale_shape_manual(name = "", values = c(0:7)) + scale_color_manual(name = "",
  values = rainbow(8)) + stat_ellipse() + xlab(label.x) + ylab(label.y)
figPCAa
```

## Discrimination between populations

```
# use equal prior probability for all groups
n.gr <- length(unique(PCA.scores$group))  # number of groups
MX.lin.cva <- CVA(PCA.scores[, pcNames], PCA.scores$group, rounds = 10000, cv = TRUE,
  prior = rep(1/n.gr, n.gr))

# Mahalanobis distances between groups
knitr::kable(as.data.frame(as.matrix(MX.lin.cva$Dist$GroupdistMaha)), digits = 3)
```

|  | lineage A | lineage C | lineage M | lineage O | Argentina | Ecuador | Mexico | USA |
| --- | --- | --- | --- | --- | --- | --- | --- | --- |
| lineage A | 0.00 | 8.31 | 7.72 | 6.36 | 6.17 | 7.78 | 7.30 | 6.94 |
| lineage C | 8.31 | 0.00 | 10.52 | 9.02 | 8.19 | 8.74 | 9.47 | 7.31 |
| lineage M | 7.72 | 10.52 | 0.00 | 9.93 | 9.56 | 10.43 | 11.09 | 10.56 |
| lineage O | 6.36 | 9.02 | 9.93 | 0.00 | 7.98 | 9.42 | 11.50 | 8.11 |
| Argentina | 6.17 | 8.19 | 9.56 | 7.98 | 0.00 | 6.16 | 7.75 | 2.45 |
| Ecuador | 7.78 | 8.74 | 10.43 | 9.42 | 6.16 | 0.00 | 7.91 | 5.95 |
| Mexico | 7.30 | 9.47 | 11.09 | 11.50 | 7.75 | 7.91 | 0.00 | 8.01 |
| USA | 6.94 | 7.31 | 10.56 | 8.11 | 2.45 | 5.95 | 8.01 | 0.00 |

```
knitr::kable(as.data.frame(as.matrix(MX.lin.cva$Dist$probsMaha)), digits = 5, format.args = list(scientific = TRUE))
```

|  | lineage A | lineage C | lineage M | lineage O | Argentina | Ecuador | Mexico | USA |
| --- | --- | --- | --- | --- | --- | --- | --- | --- |
| lineage A | 0e+00 | 1e-04 | 1e-04 | 1e-04 | 1.00e-04 | 1e-04 | 1e-04 | 1.00e-04 |
| lineage C | 1e-04 | 0e+00 | 1e-04 | 1e-04 | 1.00e-04 | 1e-04 | 1e-04 | 1.00e-04 |
| lineage M | 1e-04 | 1e-04 | 0e+00 | 1e-04 | 1.00e-04 | 1e-04 | 1e-04 | 1.00e-04 |
| lineage O | 1e-04 | 1e-04 | 1e-04 | 0e+00 | 1.00e-04 | 1e-04 | 1e-04 | 1.00e-04 |
| Argentina | 1e-04 | 1e-04 | 1e-04 | 1e-04 | 0.00e+00 | 1e-04 | 1e-04 | 8.35e-01 |
| Ecuador | 1e-04 | 1e-04 | 1e-04 | 1e-04 | 1.00e-04 | 0e+00 | 1e-04 | 1.00e-04 |
| Mexico | 1e-04 | 1e-04 | 1e-04 | 1e-04 | 1.00e-04 | 1e-04 | 0e+00 | 1.00e-04 |
| USA | 1e-04 | 1e-04 | 1e-04 | 1e-04 | 8.35e-01 | 1e-04 | 1e-04 | 0.00e+00 |

```
PCoA <- cmdscale(MX.lin.cva$Dist$GroupdistMaha)
colnames(PCoA) <- c("PCo1", "PCo2")
ggplot(data = as.data.frame(PCoA), aes(x = PCo1, y = PCo2)) + geom_point() + geom_label(label = rownames(PCoA),
  nudge_x = 0, nudge_y = 0.6) + xlim(c(-6.5, 5))
```

```
# ggsave('PCoA.pdf', width = 170, height = 107, units = 'mm')
# ggsave('PCoA.png', width = 170, height = 107, units = 'mm')

MX.lin.scores <- as.data.frame(MX.lin.cva$CVscores)
# rename variable names from CV to LD otherwise use `CV 1`
colnames(MX.lin.scores) <- gsub("CV ", "LD", colnames(MX.lin.scores))
MX.lin.scores$group <- PCA.scores$group
rownames(MX.lin.scores) <- rownames(PCA.scores)

figPCAb <- ggplot(MX.lin.scores, aes(x = LD1, y = LD2, shape = group, color = group)) +
  geom_point() + scale_shape_manual(name = "", values = c(0:7)) + scale_color_manual(name = "",
  values = rainbow(8)) + stat_ellipse()

figPCAb
```

```
ggarrange(figPCAa, figPCAb, labels = c("a", "b"), font.label = list(size = 12, face = "bold"),
  ncol = 2, nrow = 1, common.legend = TRUE, legend = "bottom")
```

```
# ggsave('pop-PCA.pdf', width = 170, height = 107, units = 'mm')
# ggsave('pop-PCA.png', width = 170, height = 107, units = 'mm')

CVA.class <- typprobClass(MX.lin.cva$CVscores, groups = as.factor(PCA.scores$group),
  outlier = 0)
print(CVA.class)
```

```
 cross-validated classification results in frequencies
           
            Argentina Ecuador lineage A lineage C lineage M lineage O Mexico
  lineage A         1       0        84         0         0         0      0
  lineage C         0       0         0        37         0         0      0
  lineage M         0       0         0         0        16         0      0
  lineage O         0       0         1         0         0        48      0
  Argentina        13       0         0         0         0         0      0
  Ecuador           1      73         0         0         0         0      0
  Mexico            0       0         0         0         0         0    245
  USA               1       0         0         0         0         0      0
           
            USA
  lineage A   0
  lineage C   0
  lineage M   0
  lineage O   0
  Argentina   5
  Ecuador     0
  Mexico      0
  USA         9


 cross-validated classification result in %
           
            Argentina  Ecuador lineage A lineage C lineage M lineage O   Mexico
  lineage A    1.1765   0.0000   98.8235    0.0000    0.0000    0.0000   0.0000
  lineage C    0.0000   0.0000    0.0000  100.0000    0.0000    0.0000   0.0000
  lineage M    0.0000   0.0000    0.0000    0.0000  100.0000    0.0000   0.0000
  lineage O    0.0000   0.0000    2.0408    0.0000    0.0000   97.9592   0.0000
  Argentina   72.2222   0.0000    0.0000    0.0000    0.0000    0.0000   0.0000
  Ecuador      1.3514  98.6486    0.0000    0.0000    0.0000    0.0000   0.0000
  Mexico       0.0000   0.0000    0.0000    0.0000    0.0000    0.0000 100.0000
  USA         10.0000   0.0000    0.0000    0.0000    0.0000    0.0000   0.0000
           
                 USA
  lineage A   0.0000
  lineage C   0.0000
  lineage M   0.0000
  lineage O   0.0000
  Argentina  27.7778
  Ecuador     0.0000
  Mexico      0.0000
  USA        90.0000


 overall classification accuracy: 98.31461 %

 Kappa statistic: 0.97753
```

```
pTab <- as.data.frame(CVA.class$probs)
sums <- rowSums(pTab)
idLong <- pTab/sums
idLong$sample <- rownames(xySample)
idLong <- melt(idLong, id.vars = c("sample"))
idLong$gr <- substr(idLong$sample, 1, 2)  # short 2 letter name
idLong$gr <- gsub("-", "", idLong$gr)
idLong$gr <- factor(idLong$gr, levels = c("A", "C", "M", "O", "AR", "EC", "MX", "US"))  #specify order

ggplot(idLong, aes(sample, value, fill = variable)) + geom_col(width = 1) + scale_fill_manual(values = rainbow(8),
  name = "") + facet_grid(~gr, switch = "x", scales = "free", space = "free") +
  theme_minimal() + labs(x = NULL, y = NULL) + scale_y_continuous(expand = c(0,
  0)) + scale_x_discrete(expand = expand_scale(add = 1)) + theme(panel.spacing.x = unit(0.1,
  "lines"), axis.text.x = element_blank(), panel.grid = element_blank(), legend.position = "bottom")
```

## MANOVA of populations

```
MANOVA.all <- manova(as.matrix(cbind(PCA.scores[, pcNames])) ~ group, PCA.scores)
summary(MANOVA.all)
```

```
           Df Pillai approx F num Df den Df Pr(>F)    
group       7   4.21     22.2    238   3493 <2e-16 ***
Residuals 526                                         
---
Signif. codes:  0 '***' 0.001 '**' 0.01 '*' 0.05 '.' 0.1 ' ' 1
```

# Clustering

```
summary(PCA)  # first 18 PCs explains 95% of variance
```

```
Importance of components:
                           PC1     PC2     PC3     PC4     PC5     PC6     PC7
Standard deviation     0.00822 0.00651 0.00598 0.00507 0.00458 0.00373 0.00344
Proportion of Variance 0.22321 0.14024 0.11828 0.08486 0.06948 0.04591 0.03907
Cumulative Proportion  0.22321 0.36345 0.48173 0.56659 0.63607 0.68199 0.72105
                           PC8     PC9    PC10    PC11    PC12    PC13   PC14
Standard deviation     0.00338 0.00319 0.00291 0.00274 0.00262 0.00259 0.0023
Proportion of Variance 0.03767 0.03356 0.02802 0.02473 0.02276 0.02215 0.0174
Cumulative Proportion  0.75872 0.79228 0.82030 0.84503 0.86780 0.88995 0.9074
                          PC15    PC16    PC17    PC18    PC19    PC20    PC21
Standard deviation     0.00202 0.00186 0.00168 0.00159 0.00149 0.00134 0.00124
Proportion of Variance 0.01355 0.01145 0.00929 0.00837 0.00734 0.00589 0.00507
Cumulative Proportion  0.92092 0.93237 0.94166 0.95003 0.95737 0.96326 0.96834
                          PC22    PC23    PC24    PC25     PC26     PC27
Standard deviation     0.00122 0.00116 0.00106 0.00103 0.000923 0.000856
Proportion of Variance 0.00490 0.00449 0.00368 0.00351 0.002820 0.002420
Cumulative Proportion  0.97323 0.97772 0.98140 0.98491 0.987730 0.990150
                           PC28     PC29     PC30     PC31     PC32     PC33
Standard deviation     0.000837 0.000805 0.000728 0.000636 0.000575 0.000447
Proportion of Variance 0.002320 0.002140 0.001750 0.001340 0.001090 0.000660
Cumulative Proportion  0.992470 0.994610 0.996360 0.997700 0.998790 0.999450
                           PC34     PC35     PC36     PC37     PC38
Standard deviation     0.000407 5.09e-17 4.29e-17 1.64e-17 1.45e-17
Proportion of Variance 0.000550 0.00e+00 0.00e+00 0.00e+00 0.00e+00
Cumulative Proportion  1.000000 1.00e+00 1.00e+00 1.00e+00 1.00e+00
```

```
pcNames1_18 <- paste0("PC", 1:18)  # create principal components names for PC1:PC18

# clustering of first 18 Pcs, range of possible clusters 1:9
clust <- Mclust(as.matrix(PCA.scores[, pcNames1_18]), G = 1:9)
summary(clust)
```

```
---------------------------------------------------- 
Gaussian finite mixture model fitted by EM algorithm 
---------------------------------------------------- 

Mclust VEI (diagonal, equal shape) model with 5 components: 

 log-likelihood   n  df   BIC   ICL
          42012 534 116 83296 83207

Clustering table:
  1   2   3   4   5 
246 167  60  28  33
```

```
plot(clust, what = "BIC")  # plot Bayesian Information Criterion
```

```
m.best <- dim(clust$z)[2]
cat("Model-based optimal number of clusters:", m.best, "\n")
```

```
Model-based optimal number of clusters: 5
```

```
cluTab <- table(PCA.scores$group, clust$classification)
colnames(cluTab) <- paste0("cluster ", colnames(cluTab))
cluTab  # frequencies in clusters
```

```
            cluster 1 cluster 2 cluster 3 cluster 4 cluster 5
  lineage A        17        68         0         0         0
  lineage C         0         4         0         0        33
  lineage M         0        16         0         0         0
  lineage O         0        49         0         0         0
  Argentina         0         0         0        18         0
  Ecuador           4        11        59         0         0
  Mexico          225        19         1         0         0
  USA               0         0         0        10         0
```

```
proportions(cluTab, margin = 1)  # proportions within populations
```

```
            cluster 1 cluster 2 cluster 3 cluster 4 cluster 5
  lineage A   0.20000   0.80000   0.00000   0.00000   0.00000
  lineage C   0.00000   0.10811   0.00000   0.00000   0.89189
  lineage M   0.00000   1.00000   0.00000   0.00000   0.00000
  lineage O   0.00000   1.00000   0.00000   0.00000   0.00000
  Argentina   0.00000   0.00000   0.00000   1.00000   0.00000
  Ecuador     0.05405   0.14865   0.79730   0.00000   0.00000
  Mexico      0.91837   0.07755   0.00408   0.00000   0.00000
  USA         0.00000   0.00000   0.00000   1.00000   0.00000
```

```
proportions(cluTab, margin = 2)  # proportions within clusters
```

```
            cluster 1 cluster 2 cluster 3 cluster 4 cluster 5
  lineage A    0.0691    0.4072    0.0000    0.0000    0.0000
  lineage C    0.0000    0.0240    0.0000    0.0000    1.0000
  lineage M    0.0000    0.0958    0.0000    0.0000    0.0000
  lineage O    0.0000    0.2934    0.0000    0.0000    0.0000
  Argentina    0.0000    0.0000    0.0000    0.6429    0.0000
  Ecuador      0.0163    0.0659    0.9833    0.0000    0.0000
  Mexico       0.9146    0.1138    0.0167    0.0000    0.0000
  USA          0.0000    0.0000    0.0000    0.3571    0.0000
```

```
pTab <- as.data.frame(clust$z)  # probabilities of belonging to clusters
colnames(pTab) <- gsub("V", "cluster ", colnames(pTab))
sums <- rowSums(pTab)
pTabLong <- pTab/sums
pTabLong$sample <- rownames(pTabLong)
pTabLong <- melt(pTabLong, id.vars = c("sample"))
pTabLong$gr <- substr(pTabLong$sample, 1, 2)  # short 2 letter name
pTabLong$gr <- gsub("-", "", pTabLong$gr)  # remove '-'
pTabLong$gr <- factor(pTabLong$gr, levels = c("A", "C", "M", "O", "AR", "EC", "MX",
  "US"))  #specify order

figAdmClu <- ggplot(pTabLong, aes(sample, value, fill = variable)) + geom_col(width = 1) +
  scale_fill_manual(values = rainbow(5), name = "") + facet_grid(~gr, switch = "x",
  scales = "free", space = "free") + theme_minimal() + labs(x = NULL, y = NULL) +
  scale_y_continuous(expand = c(0, 0)) + theme(panel.spacing.x = unit(0.2, "lines"),
  axis.text.x = element_blank(), legend.position = "top")
figAdmClu
```

# Classification of samples as lineages

The data are related to study by Nawrocka et al., (2018a). The identification data are available at Zenodo

```
idData <- xml2gmLdaData("https://zenodo.org/record/14054009/files/apis-mellifera-lineage.dw.xml")
id <- gmLdaData2id(idData, xySample[, xyNames], average = FALSE)
tab <- table(id$id$group, xySample$group)
tab
```

```
    lineage A lineage C lineage M lineage O Argentina Ecuador Mexico USA
  A        85         0         0         0        17      70    242   9
  C         0        37         0         0         1       3      3   1
  M         0         0        16         0         0       0      0   0
  O         0         0         0        49         0       1      0   0
```

```
proportions(tab, margin = 2)
```

```
    lineage A lineage C lineage M lineage O Argentina Ecuador Mexico    USA
  A    1.0000    0.0000    0.0000    0.0000    0.9444  0.9459 0.9878 0.9000
  C    0.0000    1.0000    0.0000    0.0000    0.0556  0.0405 0.0122 0.1000
  M    0.0000    0.0000    1.0000    0.0000    0.0000  0.0000 0.0000 0.0000
  O    0.0000    0.0000    0.0000    1.0000    0.0000  0.0135 0.0000 0.0000
```

```
pTab <- as.data.frame(id$P)
sums <- rowSums(pTab)
pTabLong <- pTab/sums
pTabLong$sample <- rownames(pTabLong)
pTabLong <- melt(pTabLong, id.vars = c("sample"))
pTabLong$gr <- substr(pTabLong$sample, 1, 2)  # short 2 letter name
pTabLong$gr <- gsub("-", "", pTabLong$gr)  # remove '-'
pTabLong$gr <- factor(pTabLong$gr, levels = c("A", "C", "M", "O", "AR", "EC", "MX",
  "US"))  #specify order

figAdmLin <- ggplot(pTabLong, aes(sample, value, fill = variable)) + geom_col(width = 1) +
  scale_fill_manual(values = rainbow(5), name = "") + facet_grid(~gr, switch = "x",
  scales = "free", space = "free") + theme_minimal() + labs(x = NULL, y = NULL) +
  scale_y_continuous(expand = c(0, 0)) + theme(panel.spacing.x = unit(0.2, "lines"),
  axis.text.x = element_blank(), legend.position = "top")
figAdmLin
```

```
ggarrange(figAdmClu, figAdmLin, labels = c("a", "b"), font.label = list(size = 12,
  face = "bold"), ncol = 1, nrow = 2)
```

```
# ggsave('admixture.pdf', width = 170, height = 107, units = 'mm')
# ggsave('admixture.png', width = 170, height = 107, units = 'mm')

# classification results per country
idAll <- id$id
idAll$gr <- substr(rownames(idAll), 1, 2)
idAll <- idAll[!idAll$gr %in% c("A-", "C-", "M-", "O-"), ]
idAll <- cbind(idAll, geoDataSample)

# Mexico
idMX <- idAll[idAll$country == "Mexico", ]
table(idMX$group)
```

```
  A   C 
242   3
```

```
ggplot(data = world) + geom_sf(fill = NA) + geom_jitter(data = idMX, aes(x = longitude,
  y = latitude, colour = group), width = 0.3, height = 0.3, size = 1) + coord_sf(xlim = c(x.min,
  x.max), ylim = c(y.min, y.max)) + theme(legend.position = "bottom")
```

```
# Argentina
idAR <- idAll[idAll$country == "Argentina", ]
table(idAR$group)
```

```
 A  C 
17  1
```

```
ggplot(data = world) + geom_sf(fill = NA) + geom_point(data = idAR, aes(x = longitude,
  y = latitude, colour = group), size = 1) + coord_sf(xlim = c(-50, -70), ylim = c(-20,
  -40)) + theme(legend.position = "bottom")
```

```
# USA
idUS <- idAll[idAll$country == "USA", ]
table(idUS$group)
```

```
A C 
9 1
```

```
ggplot(data = world) + geom_sf(fill = NA) + geom_point(data = idUS, aes(x = longitude,
  y = latitude, colour = group), size = 1) + coord_sf(xlim = c(-130, -110), ylim = c(20,
  40)) + theme(legend.position = "bottom")
```

```
# Ecuador
idEC <- idAll[idAll$country == "Ecuador", ]
table(idEC$group)
```

```
 A  C  O 
70  3  1
```

```
ggplot(data = world) + geom_sf(fill = NA) + geom_jitter(data = idEC, aes(x = longitude,
  y = latitude, colour = group), width = 0.05, height = 0.05, size = 1) + coord_sf(xlim = c(-79,
  -78.5), ylim = c(-2, -1)) + theme(legend.position = "bottom")
```

# References

Calfee, E., Agra, M. N., Palacio, M. A., Ramírez, S. R., & Coop, G. (2020). Selection and hybridization shaped the rapid spread of African honey bee ancestry in the Americas. PLoS genetics, 16(10), e1009038. https://doi.org/10.1371/journal.pgen.1009038

Calfee, E., Agra, M. N., Palacio, M. A., Ramírez, S. R., & Coop, G. (2020). Apis mellifera wing images (Africanized honey bees) [Dataset]. Dryad. https://doi.org/10.25338/B8T032

Masaquiza, D., & Arenal, A. (2024). Collection of images and raw coordinates of honey bee (Apis mellifera) wings from the central highlands of Ecuador. Zenodo. https://doi.org/10.5281/zenodo.13340594

Masaquiza, D., Rodríguez, L.C., Zapata, J., Monar, J., Vaca, M., Porrini, L., Eguaras, M., Daniele, M., Romero, D., Arenal, A. (2024). Use of Wing Geometric Morphometric Analysis and mtDNA to Identify Africanization of Apis mellifera in the Central Highlands of Ecuador. Insects, 15, 628. https://doi.org/10.3390/insects15080628

Nawrocka, A., Kandemir, İ., Fuchs, S., & Tofilski, A. (2018). Computer software for identification of honey bee subspecies and evolutionary lineages. Apidologie, 49(2), 172-184. https://doi.org/10.1007/s13592-017-0538-y

Nawrocka, A., Kandemir, İ., Fuchs, S., & Tofiilski, A. (2018). Dataset: Computer software for identification of honey bee subspecies and evolutionary lineages. Apidologie, 49, 172–184. https://doi.org/10.5281/zenodo.7567336

Payró de la Cruz, E., Valencia Domínguez, M., Ramos Reyes, R., & Tofilski, A. (2024). Fore wings of honey bees (*Apis mellifera*) from Tabasco, Mexico [Data set]. Zenodo. https://doi.org/10.5281/zenodo.13884732

Tofilski, A. (2025). Measurements of honey bee (*Apis mellifera*) wings originally provided by Calfee et al. (2020) [Data set]. Zenodo. https://doi.org/10.5281/zenodo.14604479

Tofilski, A. (2025). Measurements of honey bee (*Apis mellifera*) wings originally provided by Masaquiza et al. (2024) [Data set]. Zenodo. https://doi.org/10.5281/zenodo.14604363

# Information about session

```
sessionInfo()
```

```
R version 4.3.2 (2023-10-31 ucrt)
Platform: x86_64-w64-mingw32/x64 (64-bit)
Running under: Windows 11 x64 (build 22000)

Matrix products: default


locale:
[1] LC_COLLATE=Polish_Poland.utf8  LC_CTYPE=Polish_Poland.utf8   
[3] LC_MONETARY=Polish_Poland.utf8 LC_NUMERIC=C                  
[5] LC_TIME=Polish_Poland.utf8    

time zone: Europe/Warsaw
tzcode source: internal

attached base packages:
[1] stats     graphics  grDevices utils     datasets  methods   base     

other attached packages:
 [1] reshape2_1.4.4      ggpubr_0.6.0        ggspatial_1.1.9    
 [4] raster_3.6-26       sp_2.1-1            rnaturalearth_0.3.4
 [7] ggplot2_3.5.1       mclust_6.1.1        dplyr_1.1.4        
[10] IdentiFlyR_0.1.1    Morpho_2.11         geomorph_4.0.6     
[13] Matrix_1.6-1.1      rgl_1.2.1           RRPP_1.4.0         

loaded via a namespace (and not attached):
 [1] tidyselect_1.2.0        farver_2.1.1            fastmap_1.1.1          
 [4] tweenr_2.0.2            digest_0.6.33           lifecycle_1.0.4        
 [7] sf_1.0-14               terra_1.7-55            bezier_1.1.2           
[10] magrittr_2.0.3          compiler_4.3.2          rlang_1.1.2            
[13] tools_4.3.2             utf8_1.2.4              yaml_2.3.7             
[16] knitr_1.45              ggsignif_0.6.4          labeling_0.4.3         
[19] htmlwidgets_1.6.3       scatterplot3d_0.3-44    curl_5.1.0             
[22] classInt_0.4-10         xml2_1.3.6              plyr_1.8.9             
[25] shapes_1.2.7            abind_1.4-5             KernSmooth_2.23-22     
[28] withr_2.5.2             purrr_1.0.2             polyclip_1.10-6        
[31] grid_4.3.2              fansi_1.0.5             e1071_1.7-13           
[34] colorspace_2.1-0        scales_1.3.0            iterators_1.0.14       
[37] MASS_7.3-60             cli_3.6.1               rmarkdown_2.25         
[40] generics_0.1.3          rstudioapi_0.15.0       httr_1.4.7             
[43] Rvcg_0.22.1             ggforce_0.4.1           DBI_1.1.3              
[46] ape_5.7-1               proxy_0.4-27            stringr_1.5.1          
[49] parallel_4.3.2          formatR_1.14            base64enc_0.1-3        
[52] vctrs_0.6.4             minpack.lm_1.2-4        jsonlite_1.8.7         
[55] carData_3.0-5           car_3.1-2               ggrepel_0.9.4          
[58] rstatix_0.7.2           jpeg_0.1-10             foreach_1.5.2          
[61] tidyr_1.3.0             units_0.8-4             colorRamps_2.3.1       
[64] glue_1.6.2              codetools_0.2-19        cowplot_1.1.1          
[67] stringi_1.8.2           gtable_0.3.4            munsell_0.5.0          
[70] tibble_3.2.1            pillar_1.9.0            htmltools_0.5.7        
[73] rnaturalearthdata_0.1.0 R6_2.5.1                doParallel_1.0.17      
[76] evaluate_0.23           lattice_0.22-6          backports_1.4.1        
[79] broom_1.0.5             class_7.3-22            Rcpp_1.0.11            
[82] gridExtra_2.3           nlme_3.1-163            xfun_0.41              
[85] pkgconfig_2.0.3
```
